# Editorial: Leptin, obesity and diet at a glance

**DOI:** 10.3389/fendo.2022.1073850

**Published:** 2022-11-09

**Authors:** Deanne H. Hryciw, Claire J. Stocker, Margaret J. Morris, Massimiliano Caprio

**Affiliations:** ^1^ School of Environment and Science, Griffith University, Nathan, QLD, Australia; ^2^ Griffith Institute of Drug Discovery, Griffith University, Nathan, QLD, Australia; ^3^ Aston Medical School, Aston University, Birmingham, United Kingdom; ^4^ School of Biomedical Sciences, University of New South Wales (UNSW), Sydney, NSW, Australia; ^5^ Laboratory of Cardiovascular Endocrinology, Istituto di Ricovero e Cura a Carattere Scientifico (IRCCS) San Raffaele, Rome, Italy; ^6^ Department of Human Sciences and Promotion of the Quality of Life, San Raffaele Roma Open University, Rome, Italy

**Keywords:** leptin, obesity, diet, genotypes, leptin resistance, leptin receptor, satiety

The adipokine leptin, a product of the *ob* gene, was identified in 1994. Since that time there has been significant research focused on leptin due to its central role in maintaining hunger and satiety, and its strong influence in obesity and associated pathophysiological changes. More recent research has investigated the link between leptin and diet, and how dietary intake has the capacity to control leptin expression and leptin signalling. This Research Topic explores the links between leptin, obesity and diet, with an emerging theme focused on genetic modulators of leptin expression and function. The Research topic contains a review and mini-review manuscript, three original research manuscripts and a case report. Collectively, they highlight the role of diet and genetics in controlling leptin function and dysfunction.

The interconnection between leptin, diet and leptin resistance is reviewed in the mini-review manuscript by Mendoza-Herrera et al. Leptin resistance is enhanced by a diet rich in fat, carbohydrates, fructose and sucrose, and low in protein. Reversal of leptin resistance has been suggested by the consumption of energy-restricted diets. With their review, Mendoza-Herrera et al. acknowledge the limitations in previous studies, with methodological heterogeneity as a key barrier to this area of research. Another significant limitation in the field is represented by the lack of a universally accepted definition of leptin resistance. Mendoza-Herrera et al. propose that this is key in designing interventions, such as leptin sensitizers, and therapeutic application of individualised diets based on genotypic variability.

The review by Obradovic et al., further discusses the role of leptin in obesity. These authors highlight the mechanisms by which leptin is released into the circulation and the molecular signalling pathways activated in response to leptin receptor agonism. Obradovic et al. note the advances in therapeutic targets associated with leptin resistance and summarise the recent findings in combining leptin and a leptin sensitizer as a potential anti-obesity therapeutic. Critically, Obradovic et al. comment that the focus of future research should be on leptin regulation at the whole-body level, which could lead to the development of therapeutics which may reverse leptin resistance.

The first original research report, by Galiniak et al., focused on the hormones that regulate appetite in people with cystic fibrosis (CF). Compared to age and sex matched healthy controls, people with CF had reduced serum levels of ghrelin and agouti-signalling protein, but no difference in putative peptide YY and alpha-melanocyte-stimulating hormone level, all hormones critical for appetite regulation. Importantly, there was no difference in hormone levels between females and males with CF, nor between the different genotypes. Further analysis indicated that ghrelin was negatively correlated with age, body mass index, and C-reactive protein and putative peptide YY was negatively associated with age. This novel research identified new targets that should be investigated in the regulation of body weight in people with CF.

The original manuscript by Sket et al. investigated genetic variability in genes associated with leptin-melanocortin signalling pathway that are linked to childhood obesity. Sket et al. identified genetic variations in the LEPR, PCSK1, POMC, MC3R and MC4R genes which were linked to obesity in childhood. These variants would result in approximately 7.6 kg increase in girls and 8.4 kg increase in body weight in boys by the age of 18 years. Sket et al. propose that the genetic predisposition to obesity should be determined to potentially reduce the societal burden and improve the clinical management in children with obesity.

In the third original manuscript by Wargent et al., the authors investigated the influence of the mouse strain in GPR17 knockout (-/-) mice and obesity. Wargent et al. determined that GPR17 -/- mice on a background of the 129 strain, displayed increased expression of uncoupling protein-1 in white adipose tissue, lower body weight and fat content, reduced plasma leptin, non-esterified fatty acids and triglycerides, and resistance to high fat diet-induced glucose intolerance, compared with the C57Bl/6 strain. Further, Wargent et al. demonstrated that administration of leptin did not suppress the increased food intake in GPR17 -/- mice of the 129 strain, compared with wildtype mice. In GPR17 -/- mice, developed in the C57Bl/6 strain, the only change was a reduction in body weight compared to wildtype. Mechanistically, the lack of GPR17 in the 129 strain, as proposed by Wargent et al., leads to an increase in sympathetic activity not observed in the C57Bl/6 strain. Thus, when data from mouse models are exploited to justify investigations into novel therapeutic targets for obesity, it is critical that the backgrounds of mouse strains are carefully taken into consideration.

The final manuscript of this Research Topic by Lambadiari et al., which investigated the potential to improve outcomes in a patient with familial partial lipodystrophy type 3. This rare autosomal disorder is caused by a mutation in the peroxisome proliferator activated receptor gamma gene, resulting in subnormal leptin secretion among other metabolic complications. In this patient, there was a misdiagnosis of Type 1 Diabetes Mellitus, and treatment with the synthetic analogue of leptin, metreleptin, improved glycaemic control (assessed *via* HbA1c concentrations) and reduced fasting plasma triglyceride concentrations. This study highlights the importance of understanding the underlying pathological change in patients, to ensure that correct therapeutic programs could be developed.

The contributions from this Research Topic provide more insight into the molecular pathways that control leptin and leptin signalling, and the effects of genetics, diet and the role of leptin in satiety. In light of the growing morbidity and mortality associated with obesity, this is particularly relevant given the paucity of safe and effective therapeutic approaches to manage obesity (1).

## Author contributions

DH drafted the first draft of the manuscript. All authors provided feedback and accepted the final edited version of the manuscript.

## Funding

MJM: National Health and Medical Research Council of Australia (#1126929 and #1161418). MC: Italian Ministry of Health [ricerca corrente].

## Conflict of interest

The authors declare that the research was conducted in the absence of any commercial or financial relationships that could be construed as a potential conflict of interest.

## Publisher’s note

All claims expressed in this article are solely those of the authors and do not necessarily represent those of their affiliated organizations, or those of the publisher, the editors and the reviewers. Any product that may be evaluated in this article, or claim that may be made by its manufacturer, is not guaranteed or endorsed by the publisher.
